# A Prospective Pilot Study to Investigate Whether Donor‐Derived Cell‐Free DNA Can Be Used as a Biomarker of Recurrent IgA Nephropathy Post–Kidney Transplantation

**DOI:** 10.1111/iji.70010

**Published:** 2025-08-28

**Authors:** Judith Owusuwaah Asiedu‐Basoah, Stephen Weston, Jonathan Barratt, Justyna Szklarzewicz, Paul Dunn

**Affiliations:** ^1^ Transplant Laboratory University Hospitals of Leicester Leicester UK; ^2^ The University of Manchester Manchester UK; ^3^ Department of Cardiovascular Sciences University of Leicester Leicester UK; ^4^ Renal Research Department University Hospitals of Leicester Leicester UK; ^5^ TBGBiotechnology Taipei Taiwan

**Keywords:** AlloSeq cfDNA assay, donor‐derived cell‐free DNA (dd‐cfDNA), kidney transplantation, non‐invasive biomarker, recurrent IgA nephropathy

## Abstract

IgA nephropathy (IgAN) is one of the most prevalent glomerulonephropathies and commonly leads to kidney failure. The recurrence of IgAN following transplantation remains a significant concern. Currently, detecting IgAN recurrence requires a kidney biopsy, highlighting the need for non‐invasive biomarkers such as donor‐derived cell‐free DNA (dd‐cfDNA) to aid in early detection. This prospective pilot study aims to evaluate dd‐cfDNA as a non‐invasive biomarker for detecting IgAN recurrence post–kidney transplantation. Specifically, the study seeks to compare %dd‐cfDNA levels in transplanted patients with and without IgAN recurrence and correlate these levels with other kidney function parameters. A total of 32 patients with histologically confirmed IgAN were enrolled, including those with documented IgAN recurrence post‐transplantation and those without recurrence. Plasma samples were collected and processed using the AlloSeq cfDNA kit to quantify relative %dd‐cfDNA levels. Kidney function parameters, including estimated glomerular filtration rate (eGFR) and proteinuria, were also assessed. The study found no significant difference in %dd‐cfDNA levels between transplanted patients with IgAN recurrence and those without recurrence (median 0.37% [IQR 0.28%–3.53%] vs. median 0.42% [IQR 0.15%–0.84%], *p =* 0.67). Also, %dd‐cfDNA (AUC = 0.57 [95% CI 0.28–0.85], *p =* 0.64) failed to effectively discriminate IgAN recurrence compared to traditional kidney function parameters such as proteinuria (AUC = 0.96 [95% CI 0.87–1.00], *p =* 0.002) and eGFR (AUC = 0.74 [95% CI 0.47–1.00], *p =* 0.09). Relative (%) dd‐cfDNA alone may not be a robust biomarker for detecting IgAN recurrence post‐transplantation. While proteinuria proved a more effective indicator in this study, kidney biopsy remains the gold standard for definitive diagnosis. These findings highlight the challenges of using %dd‐cfDNA as a standalone diagnostic tool for monitoring IgAN recurrence post‐transplantation. Future research should explore larger patient cohorts and longitudinal assessments to refine the utility of dd‐cfDNA and investigate potential combination strategies with other biomarkers.

**Abbreviations**
ABMR: antibody‐mediated rejection
AUC: area under the curve
CI: confidence interval
dd‐cfDNA: donor‐derived cell‐free DNA
DSA: donor‐specific antibodies
eGFR: estimated glomerular filtration rate
Gd‐IgA1: galactose‐deficient IgA1
HLA: human leucocyte antigen
IgAN: immunoglobulin A nephropathy
IQR: interquartile range
NPV: negative predictive value
PPV: positive predictive value
ROC: receiver operating characteristic
TCMR: T‐cell‐mediated rejection

## 1 Introduction

Kidney transplantation presents a transformative intervention for individuals with kidney failure, offering a better quality of life and a longer life expectancy compared to dialysis (Strohmaier et al. 2022). In patients with IgA nephropathy (IgAN), the recurrence of IgAN in the transplanted kidney remains a concern, impacting both graft outcomes and the long‐term health of transplant recipients. Recurrent IgAN has been reported in up to about 60% of cases (Selvaskandan et al. 2020) with ∼13%–25% of patients experiencing recurrence‐related graft dysfunction within 5 years, which may contribute to graft loss in about 10%–15% of cases (Infante et al. 2020).

IgAN is characterised by an accumulation of immunoglobulin A in the glomerular mesangium, and its pathogenesis is explained by a multi‐hit hypothesis (Selvaskandan et al. 2022). Following transplantation, the allograft faces common transplant challenges such as T‐cell‐mediated rejection (TCMR) and/or the potential development of donor‐specific antibodies (DSAs), which can lead to antibody‐mediated rejection (ABMR) (Zhang 2018). In patients with IgAN, there is the additional risk of recurrent disease in the allograft, driven by the persistence of circulating IgA immune complexes post‐transplantation (Wyld and Chadban 2016). Younger recipient age, presence of DSAs at transplantation, a rapidly progressive disease course in the native kidneys and pre‐emptive transplantation are potential risk factors for IgAN recurrence (Bjørneklett et al. 2011; Rodas et al. 2020; Uffing et al. 2021).

Clinical tests performed in the assessment of graft function include evaluation of eGFR and testing for haematuria and proteinuria; however, changes in these tests are not specific for recurrence and insensitive in detecting early recurrence. Histological evidence of recurrence appears before clinical signs such as proteinuria, haematuria and change in eGFR are evident (Ortiz et al. 2012). At present, allograft biopsies are essential for diagnosing recurrence, although they are invasive and carry risks such as haematuria, perirenal haematomas, and occasionally, being inadequate for histopathological evaluation (Schwarz et al. 2005).

There is a need for non‐invasive tests to detect IgAN recurrence to aid prompt treatment and improve overall graft survival. Biomarkers such as serum total IgA, Gd‐IgA1, IgA‐soluble CD89 and IgA‐IgG complexes have been explored for this purpose, but results are inconsistent (Berthelot et al. 2015; Garnier et al. 2018; Berthoux et al. 2017; Jäger et al. 2022). Consequently, there is increasing interest in investigating alternative non‐invasive biomarkers.

Donor‐derived cell‐free DNA (dd‐cfDNA) is a promising biomarker for non‐invasive monitoring of allograft injury. Cell‐free DNA (cfDNA) is composed of small DNA fragments, released into the bloodstream and other bodily fluids following apoptosis and necrosis (Yan et al. 2021). Studies have explored the effectiveness of dd‐cfDNA in detecting rejection episodes, graft dysfunction and other complications in recipients of kidney, liver, heart and lung transplants (Cheng et al. 2021; Jang et al. 2021; Keller and Agbor‐Enoh 2021; Levitsky et al. 2022). Elevated levels of dd‐cfDNA are not exclusive to organ rejection. Organ injury triggers the release of cfDNA, indicating that various factors, including trauma, infection, or immune conditions, may contribute to increased levels of cfDNA (Goussous et al. 2020; Wan et al. 2013).

Upon implantation of an allograft, dd‐cfDNA is continuously released into the recipient's bloodstream and it is promptly cleared due to its short half‐life of approximately 30 min to 2 h (Sherwood and Weimer 2018). Consequently, dd‐cfDNA functions as a consistent and dynamic biomarker, facilitating the monitoring of trends, detection of injury and determination of its severity.

Research on dd‐cfDNA as a potential biomarker for IgAN recurrence post‐transplantation is currently limited. Akifova et al. (2023) investigated the use of dd‐cfDNA in distinguishing between ABMR and IgAN recurrence post‐transplantation. Among their ABMR cohort (*n* = 21), only one patient had IgAN as the original underlying disease. They reported that dd‐cfDNA levels in patients with ABMR were higher than those with recurrent IgAN. The researchers also found that even in instances of severe recurrent IgAN, the levels of dd‐cfDNA did not exhibit a notable increase. Although their study examined dd‐cfDNA levels in IgAN recurrence, there remains a gap in understanding dd‐cfDNA levels in transplanted patients with IgAN recurrence compared to those without recurrence.

This prospective pilot study aims to explore dd‐cfDNA as a non‐invasive marker for IgAN recurrence using the AlloSeq cfDNA kit. Dd‐cfDNA percentages will be compared between patients with and without IgAN recurrence in their allograft. In addition, correlations with key renal function parameters, proteinuria and eGFR will be analysed to evaluate dd‐cfDNA's diagnostic potential.

## 2 Materials and Methods

### 2.1 Patient Cohort

Thirty‐two patients with histologically proven IgAN as their primary renal disease were enrolled for this study at the University Hospitals of Leicester. Ethical approval was obtained from the East Midlands—Leicester South Research Ethics Committee (REC reference: 05/Q2502/80, IRAS project ID: 177442). Blood samples were obtained from the patients with prior informed consent. These samples were collected from August 2022 to November 2023.

Patients met specific eligibility criteria per the AlloSeq cfDNA assay guidelines to eliminate potential confounding factors that could affect the interpretation of cfDNA assay results. Eligible patients had not received: a transplant from an identical twin, allogeneic blood or bone marrow transplant, multi‐organ transplants from different donors and recent blood transfusions (within 1 month). Female patients were required not to be pregnant, and those with a tissue biopsy within 24–48 h prior to enrolment were also not eligible.

One patient in the non‐recurrent group and one in the recurrent group who had undergone a second transplant were included in the study cohort. Prior research supports the AlloSeq cfDNA kit's reliability in detecting dd‐cfDNA in retransplant patients, consistent with manufacturer guidelines (Mehta et al. 2019). However, without prior donor genotyping, the kit cannot discriminate which specific allograft(s) contribute to the total dd‐cfDNA measured in patients with more than one transplant.

**FIGURE 1:**
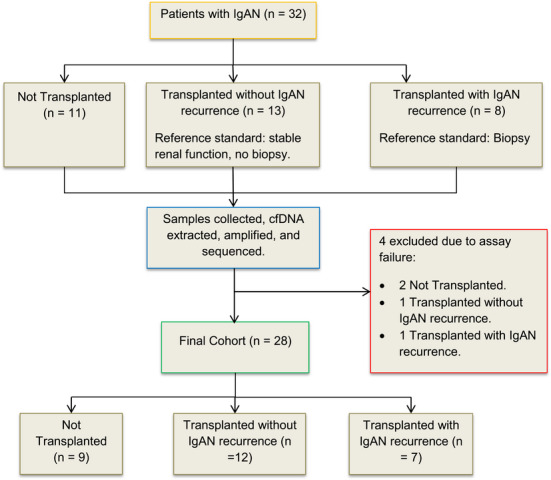
Classification of study participants into not transplanted, transplanted with recurrent IgAN and transplanted without recurrent IgAN groups. cfDNA, cell‐free DNA; IgAN, IgA nephropathy.

The cohort comprised three groups: 11 control IgAN patients with no history of kidney transplantation, 13 IgAN transplant recipients with no clinical evidence of IgAN recurrence and 8 IgAN transplant recipients with biopsy‐confirmed recurrent IgAN. It is important to note that patients in the non‐recurrent transplant group had not undergone diagnostic biopsies at the time of sample collection as they had a stable kidney function (e.g., absence of significant proteinuria or decline in eGFR), and there was no clinical indication for a biopsy. In contrast, patients in the recurrent group underwent indication biopsies prompted by clinical suspicion of recurrence, such as new‐onset or worsening proteinuria, or decline in eGFR (Figure 1).

Among transplanted patients, two without IgAN recurrence, had pre‐formed anti‐HLA DSAs; however, pre‐transplant crossmatch tests were negative. All transplanted patients were classified as having standard immunological risk based on the BSHI/BTS risk stratification guidelines (Poulton et al. 2016). In the non‐recurrent group, 8 out of 13 patients received transplants from living donors, while in the recurrent group, 2 out of 8 patients received transplants from living donors. There was no record of de novo DSAs or cases of ABMR.

The eGFR and urine protein‐to‐creatinine ratio of the patients at the time of sample collection were also collected to assess how these parameters varied with the %dd‐cfDNA of the patients.

### 2.2 Plasma Samples and cfDNA Extraction

Patients were recruited during routine outpatient clinic visits, and samples were taken after gaining informed consent.

For each patient, 8 mL of blood was drawn into two cfDNA blood collection tubes each (Streck, La Vista, NE, USA) and stored at 22°C for up to 3 days. Plasma separation was achieved by centrifuging the blood samples at 1600 × *g* for 10 min. The upper plasma layer was then transferred to new tubes and centrifuged again at 16,000 × *g* for 10 min. The resulting supernatant was transferred to fresh tubes.

cfDNA extraction was performed using 4 or 5 mL of plasma with the QIAamp Circulating Nucleic Acid Kit (Qiagen, Hilden, Germany, Cat. No., 55114) following the manufacturer's protocol. Samples not processed on the same day were stored at −80°C and thawed at room temperature before cfDNA extraction. Extracted cfDNA was quantified using the Quantus fluorometer (Promega, Wisconsin, USA), yielding concentrations ranging from 0.162 to 2.450 ng/µL.

### 2.3 Amplification and Sequencing of cfDNA

Extracted cfDNA was amplified using the AlloSeq cfDNA (RUO) kit (CareDx, Fremantle, Australia), a single multiplex polymerase chain reaction (PCR) assay that does not require prior genotyping of the recipient and donor. This assay targets 202 single‐nucleotide polymorphisms (SNPs) and leverages SNP variations to quantify the proportion of dd‐cfDNA relative to total cfDNA from a plasma sample.

Libraries containing 12–24 cfDNA samples were prepared simultaneously with an input of 2.6–8 ng. A library concentration of 20 pM with 1% PhiX control was loaded onto the Illumina MiSeq Reagent Kit v3 150 cycles sequencing cartridge (Illumina, San Diego, CA, USA) and sequenced using a MiSeq FGx Sequencing System (Illumina). The fastq files were analysed using the AlloSeq cfDNA software (v2.2.0), which automatically calculated the %dd‐cfDNA in each sample. In the absence of donor or recipient genotype information, the cfDNA that constitutes the minor fraction is designated as ‘donor‐derived’.

Four samples failed to meet the assay's internal quality control thresholds, which included sufficient informative markers, coverage uniformity, average marker coverage and total read count. These results were therefore excluded from the analysis. Of these, two were from the non‐transplanted control group, one from the non‐recurrent transplant group and one from the recurrent group (Figure 1).

### 2.4 Statistical Analysis

All statistical analyses were performed using GraphPad Prism (v10.4.1). Samples failing the AlloSeq cfDNA assay quality control thresholds (*n* = 4) were excluded from statistical analyses.

Descriptive statistics for patient age, time since transplantation, proteinuria and eGFR are presented as the median and interquartile range (IQR). For categorical variables, the data are presented as counts with corresponding percentages. The Mann–Whitney *U* test was used for inter‐group comparisons, with statistical significance set at *p* < 0.05 (two‐tailed).

A sensitivity analysis was conducted to assess the impact of excluding the transplanted patients whose samples failed sequencing. Given the lack of %dd‐cfDNA data for these patients, their %dd‐cfDNA values were imputed using the median %dd‐cfDNA of their respective groups. This imputation assumes that the missing values follow the central tendency of their cohorts and avoid introducing extreme values that could artificially skew the results. The Mann–Whitney *U* test was then repeated to compare the groups.

Previously published cut‐off threshold of 0.5% (Bu et al. 2022; Oellerich et al. 2019; Stites et al. 2020) was used to determine patients with increased levels of dd‐cfDNA (≥ 0.5%) and those with low dd‐cfDNA. This threshold was used to calculate the sensitivity, specificity, negative predictive value (NPV) and positive predictive value (PPV). Receiver operating characteristic (ROC) curve analysis was performed, and the area under the ROC curve (AUC) with 95% confidence interval (CI) was used to establish the discriminating accuracy of dd‐cfDNA, proteinuria and eGFR.

## 3 Results

### 3.1 Patient Characteristics

Of the 32 patients, 4 were excluded due to assay quality control failure leaving 9 non‐transplanted IgAN patients, 12 transplanted IgAN patients without recurrence and 7 IgAN patients with recurrence, all with a male predominance—55.6%, 83.3% and 71.4%, respectively (Table 1).

**TABLE 1:** Baseline demographic and clinical characteristics of the study cohort at time of sample collection.

Patient variables	Not transplanted (*n* = 9)	Transplanted without IgAN recurrence (*n* = 12)	Transplanted with IgAN recurrence (*n* = 7)
Sex, *n* (%)			
Male	5 (55.6)	10 (83.3)	5 (71.4)
Female	4 (44.4)	2 (16.7)	2 (28.6)
Median age (years) (IQR)	40 (27.5–57.5)	49.5 (40.5–56.8)	45 (38–57)
Donor type, *n* (%)			
Deceased	—	5 (41.7)	6 (85.7)
Living	—	7 (58.3)	1 (14.3)
HLA mismatches^a^			
A		10 (83.3)	6 (85.7)
B	—	10 (83.3)	7 (100)
DRB1		11 (91.7)	4 (57.1)
Transplant immunological risk	—	Standard	Standard
Preformed anti‐HLA DSA, *n* (%)	—	2 (16.7)	—
Median time since transplantation (months) (IQR)	—	31.5 (11.3–40.8)	97 (58–246)
Immunosuppression, *n* (%)			
CNI‐based triple immunosuppression		8 (66.7)	3 (42.9)
CNI + Antiproliferative agent	—	4 (33.3)	4 (57.1)
Kidney function			
Median eGFR (mL/min/1.73 m^2^) (IQR)	36 (25–58)	57.5 (50–70.5)	43 (19–54)
Median proteinuria^b^ (mg/mmol) (IQR)	63.2 (50–212)	5.9 (0–30.1)	160.1 (72.8–245.7)
On dialysis^c^	—	—	1
Median %dd‐cfDNA (IQR)	—	0.42 (0.15–0.84)	0.37 (0.28–3.53)

Abbreviations: anti‐HLA DSA, anti‐human leucocyte antigen donor‐specific antibodies; eGFR, estimated glomerular filtration rate; IQR, interquartile range.

^a^ HLA mismatches data given are the number of patients who has one or two HLA mismatches for HLA‐A, ‐B and ‐DRB1.

^b^ Proteinuria is defined as urine protein‐to‐creatinine ratio.

^c^ There was no proteinuria data available for the patient on dialysis.

The median age of patients without a transplant was 40 years (IQR 27.5–57.5). The ages of transplanted patients without IgAN recurrence and those with recurrence were similar (median 49.5 [IQR 40.5–56.8] vs. 45 years [IQR 38–57], *p =* 0.92). Patients experiencing IgAN recurrence had a significantly longer median time since transplantation compared to those without recurrence (median 97 [IQR 58–246] vs. 31.5 months [IQR 11.3–40.8], *p =* 0.003). Notably, 85.7% of allografts for patients with IgAN recurrence were from deceased donors, contrasting with 41.7% for those without recurrence.

The four excluded individuals were comparable to included patients in terms of age, sex and clinical characteristics (Table 2).

**TABLE 2:** Baseline demographic and clinical characteristics of patients excluded following sequencing quality control failure.

Characteristic	Patient 1	Patient 2	Patient 3	Patient 4
Group	Not transplanted	Not transplanted	Transplanted without IgAN recurrence	Transplanted with IgAN recurrence
Sex	Male	Male	Male	Male
Age (years)	53	60	44	30
Donor type	—	—	Living	Living
HLA mismatches at HLA‐A, ‐B and ‐DRB1?	—	—	Yes	Yes
Time since transplantation (months)	—	—	14	62
Immunosuppression	—	—	CNI‐based triple immunosuppression	CNI‐based triple immunosuppression
Kidney function				
eGFR (mL/min/1.73 m^2^)	33	73	53	31
Proteinuria (mg/mmol)	215.2	97.1	14.4	29.9

### 3.2 Relative (%) dd‐cfDNA Does Not Differ Between Transplanted Patients With and Without IgAN Recurrence

The relative (%) dd‐cfDNA measured using the AlloSeq cfDNA assay for transplanted patients without IgAN recurrence ranged from 0.04% to 1.83%. Measurements for those with recurrence ranged from 0.1% to 4.21%. Although patients without a transplant do not have dd‐cfDNA, the cfDNA for the patients ranged from 98.43% to 99.98% (median 99.91 [IQR 98.91–99.96]).

**FIGURE 2:**
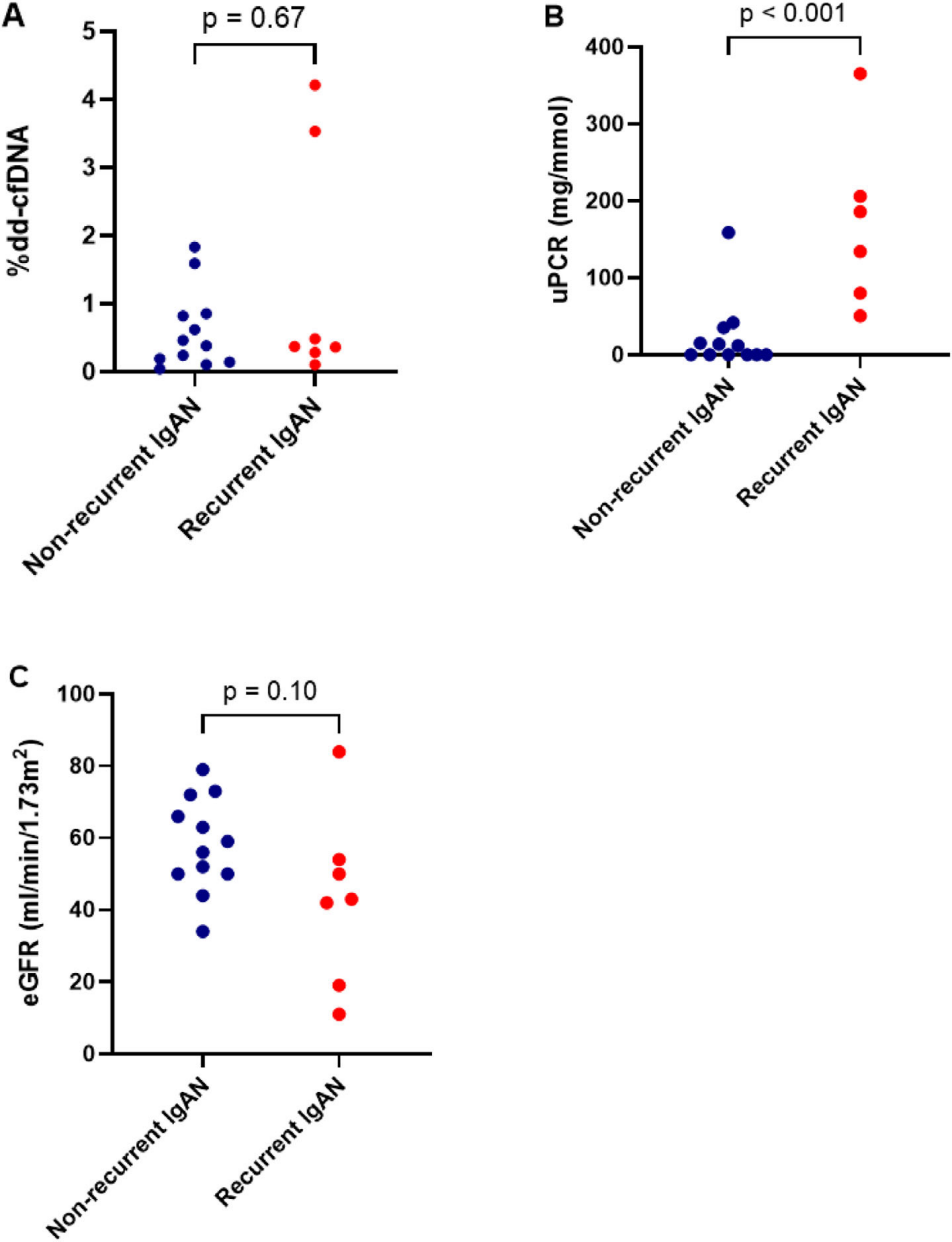
(A) %dd‐cfDNA, (B) proteinuria and (C) eGFR of transplanted patients with and without IgAN recurrence. dd‐cfDNA, donor‐derived cell‐free DNA; eGFR, estimated glomerular filtration rate.

The non‐parametric Mann–Whitney *U* test revealed that there was no significant difference in the %dd‐cfDNA levels between transplanted patients with IgAN recurrence and those without recurrence (median 0.37% [IQR 0.28%–3.53%] vs. median 0.42% [IQR 0.15%–0.84%], *p =* 0.67) (Figure 2). The %dd‐cfDNA measured for the patient with a second transplant in the recurrent group was 0.46%, while that for the patient in the non‐recurrent group was 4.21%. Including these patients did not skew the overall comparison between the two groups.

The sensitivity analysis assessing the impact of excluding transplanted patients whose samples failed AlloSeq cfDNA sequencing showed that their exclusion did not significantly alter the results. The comparison between the recurrent and non‐recurrent groups remained non‐significant (adjusted median 0.37% [IQR 0.30%–2.77%] vs. 0.42% [IQR 0.17%–0.84%], *p* = 0.82), closely matching the original analysis.

Using a previously defined %dd‐cfDNA cut‐off threshold of 0.5%, 5 (41.67%) transplanted patients without IgAN recurrence and 2 (28.57%) of those with recurrent IgAN had increased %dd‐cfDNA. At this cut‐off threshold, the test had a sensitivity of 28.6%, specificity of 58.3%, NPV of 58.3% and PPV of 28.6%.

### 3.3 Proteinuria and eGFR Are Better Discriminators of IgAN Recurrence Compared to %dd‐cfDNA

The recorded proteinuria (protein‐to‐creatinine ratio) at the time of sample collection for %dd‐cfDNA measurement was compared for pairs within the three groups using the Mann–Whitney *U* test. For patients without available proteinuria measurements due to low protein excretion (< 0.06 g/L), a value of 0 mg/mmol was assigned. This approach ensured that all patients were included in the analysis and allowed for consistent statistical comparison across the cohort.

Non‐transplanted IgAN patients exhibited higher proteinuria than transplanted patients without IgAN recurrence (median 63.20 [IQR 50–212] vs. 5.9 mg/mmol [IQR 0–30.1], *p* < 0.001). No significant difference was found between non‐transplanted IgAN patients and those with IgAN recurrence (median 63.20 [IQR 50–212] vs. 160.1 mg/mmol [IQR 72.8–245.7], *p =* 0.27). However, transplanted patients with IgAN recurrence showed higher proteinuria than those without recurrence (median 160.1 [IQR 72.8–245.7] vs. 5.9 mg/mmol [IQR 0–30.1], *p* < 0.001) (Figure 2).

The eGFR was also compared between pairs in the three groups using the Mann–Whitney *U* test. The eGFR for non‐transplanted patients was lower than transplanted patients without IgAN recurrence (median 36 [IQR 25–58] vs. 57.5 mL/min/1.73 m^2^ [IQR 50–70.5], *p* = 0.02). There was no significant difference between the eGFR of non‐transplanted patients and transplanted patients with recurrence (median 36 [IQR 25–58] vs. 43 mL/min/1.73 m^2^ [IQR 19–54], *p* = 0.90). Although transplanted patients with IgAN recurrence had a lower eGFR compared to those without recurrence, the difference did not reach statistical significance (43 [IQR 19–54] vs. 57.5 mL/min/1.73 m^2^ [IQR 50–70.5], *p* = 0.10) (Figure 2)

**FIGURE 3:**
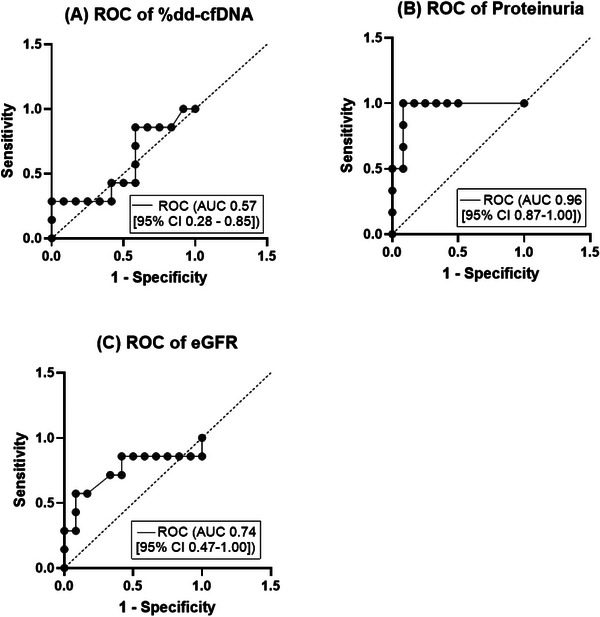
ROC curves for %dd‐cfDNA, proteinuria, and eGFR in discriminating between non‐recurrent and recurrent IgAN. (A) %dd‐cfDNA: AUC 0.57 (95% CI 0.28–0.85). (B) Proteinuria: AUC 0.96 (95% CI 0.87–1.00). (C) eGFR: AUC 0.74 (95% CI 0.47–1.00). AUC, area under the curve; CI, confidence interval; dd‐cfDNA, donor‐derived cell‐free DNA; eGFR, estimated glomerular filtration rate; ROC, receiver operating characteristic.

The ROC curve analysis showed that proteinuria (AUC = 0.96 [95% CI 0.87–1.00], *p =* 0.002) and eGFR (AUC = 0.74 [95% CI 0.47–1.00], *p =* 0.09) had superior discrimination for IgAN recurrence compared to %dd‐cfDNA (AUC = 0.57 [95% CI 0.28–0.85], *p =* 0.64) (Figure 3).

## 4 Discussion

The recurrence of IgAN in the transplanted kidney poses a significant concern for individuals with IgAN. Despite kidney biopsy remaining the gold standard for diagnosing IgAN recurrence, its invasive nature can lead to complications (Schwarz et al. 2005; Trajceska et al. 2019). Therefore, there is a critical need to identify reliable non‐invasive biomarkers for monitoring the health of transplanted organs and detecting signs of disease recurrence more easily. Dd‐cfDNA has emerged as a promising biomarker for detecting organ rejection; however, its potential in identifying disease recurrence is relatively understudied. To our knowledge, this prospective study is the first to directly compare %dd‐cfDNA levels between transplanted patients with and without IgAN recurrence.

This study observed a male predominance in all the patient groups aligning with existing literature suggesting a higher prevalence of IgAN in males (Knoop et al. 2013; O'Shaughnessy et al. 2015). Patients experiencing IgAN recurrence had a longer median time since transplantation, reflecting the slowly progressive nature of IgAN recurrence over time post‐transplantation, as noted in previous studies (Han et al. 2010; Jäger et al. 2022; Nijim et al. 2016). Unlike some studies that have shown a higher risk of recurrence in patients who received an allograft from living donors (Han et al. 2010; McDonald and Russ 2006), 85.7% of recurrent cases in our study involved deceased‐donor transplants. This finding aligns with a more recent report that found no significant difference in recurrence risk based on the type of donor used (Uffing et al. 2021).

Our findings showed no significant difference in %dd‐cfDNA levels between patients with and without IgAN recurrence (*p* = 0.67). Including patients who had received a second transplant did not skew the findings from this study. Mehta et al. (2019) observed in their study that repeat kidney transplant recipients had higher dd‐cfDNA levels compared to single kidney transplant recipients likely reflecting the combined contribution from multiple allografts. In our cohort, the retransplanted patient in the non‐recurrent group had a %dd‐cfDNA of 0.46%, closely matching the median %dd‐cfDNA for that group (0.42%). By contrast, the retransplanted patient in the recurrent group exhibited the highest %dd‐cfDNA level among all patients studied, at 4.21%. While these individual data points are notable, they did not significantly impact the group‐level comparisons.

The ROC curve (AUC 0.57 [95% CI 0.28–0.85]) indicated poor discriminatory ability, with equal false and true positives. This was further highlighted by the test having an NPV of 58.3% at a cut‐off threshold of 0.5%. At this threshold, the sensitivity and specificity of the test were also suboptimal at 28.6% and 58.3%, respectively. This indicates that the test fails to reliably identify true positive and true negative cases of IgAN recurrence. This could lead to unnecessary biopsies or missed recurrences, which can adversely affect patient outcomes.

These results are consistent with the Akifova et al. (2023) study, which reported that no difference was observed between patients with recurrent IgAN post‐transplantation and those without rejection (median 0.32% [IQR 0.24–0.41] vs. 0.30% [IQR 0.26–0.54], *p* = 0.983). In their cohort of transplanted patients without rejection (*n* = 21), only three patients had IgAN as their reported cause of end‐stage renal disease. Although their research groups were different, their results still support our findings that %dd‐cfDNA may not be a reliable biomarker for detecting IgAN recurrence.

It has been proposed that using percentages to define graft injury can be impacted by fluctuations in recipient cfDNA levels, affecting the calculation of dd‐cfDNA percentage (Goussous et al. 2020; Oellerich et al. 2019). An increase in recipient cfDNA might reduce the proportion of dd‐cfDNA, whereas a decrease in recipient cfDNA could inflate the percentage of dd‐cfDNA. In our study, we observed cfDNA levels ranging from 98.43% to 99.98% in IgAN patients who had not undergone transplantation. This wide range suggests variability in the cfDNA measurements, which can undermine the reliability of using %dd‐cfDNA as a biomarker. The variability in cfDNA levels could stem from numerous factors, including biological differences among patients, technical inconsistencies in sample handling or processing and the inherent imprecision of the assay itself.

These variations could contribute to the diminished sensitivity of the test, complicating the accurate assessment of dd‐cfDNA levels associated with IgAN recurrence post‐transplantation. Oellerich et al. (2019) reported absolute dd‐cfDNA (cp/mL) to be better than %dd‐cfDNA as a biomarker for biopsy‐proven rejection due to these variations. However, conflicting findings emerged from Akifova et al. (2023) study, where absolute dd‐cfDNA levels also failed to differentiate between recurrent IgAN and no rejection (median 11 cp/mL [IQR 7–13] vs. 12 cp/mL [7–16], *p* = .995). This suggests that neither %dd‐cfDNA nor absolute dd‐cfDNA levels are reliable indicators of IgAN recurrence.

Proteinuria and declining renal function are common clinical manifestations of recurrent IgAN, though early recurrence may present with positive biopsy findings despite no clinical signs (Avasare et al. 2017; Ortiz et al. 2012). In our study, proteinuria was comparable between non‐transplanted patients with IgAN and transplanted patients with IgAN recurrence. Interestingly, proteinuria levels were substantially higher in both non‐transplanted patients and transplanted patients with recurrence compared to transplanted patients without recurrence. This observation aligns with previous findings that increasing proteinuria is indicative of IgAN recurrence (Rodas et al. 2020; Okumi et al. 2019).

It is important to note that although proteinuria was evaluated as an index test for detecting recurrent IgAN, it was not used to define recurrence and did not contribute to the reference standard. This approach avoids incorporation bias, as the diagnostic performance of proteinuria was assessed independently of the histological criteria used to confirm recurrence. However, since proteinuria was one of the clinical factors guiding the decision to perform a biopsy, there remains the potential for selection bias, as patients without significant proteinuria or clinical indications were less likely to undergo biopsy and were therefore assumed to be non‐recurrent.

Our analysis also revealed some differences in eGFR levels among the studied cohorts. Specifically, transplanted patients with IgAN recurrence exhibited a lower eGFR compared to those without recurrence, although this did not reach statistical significance (*p* = 0.10). There was no significant difference in eGFR between non‐transplanted patients and transplanted patients with IgAN recurrence (*p* = 0.92). In addition, non‐transplanted patients displayed significantly lower eGFR compared to transplanted patients without IgAN recurrence (*p* = 0.02). These findings highlight the progressive nature of IgAN recurrence and its impact on kidney function post‐transplantation. Previous research by Jäger et al. (2022) also demonstrated an association between IgAN recurrence and declining kidney function over time, with significantly worse kidney function observed at 8 years post‐transplantation in patients with IgAN recurrence compared to those without recurrence (median eGFR 49  [IQR 29–68] vs. 60 mL/min/1.73 m^2^ [IQR 38–78]).

Despite the presence of proteinuria and a decline in kidney function during organ injury, studies have identified dd‐cfDNA as a superior indicator of organ injury (Akifova et al. 2023; Stites et al. 2020). However, these findings contrast with the results of the present study, which demonstrated that proteinuria and eGFR were more effective discriminators of recurrent IgAN. A biopsy, however, remains the gold standard for diagnosis.

Unlike reports from multiple studies which have shown a significant increase in %dd‐cfDNA in organ rejection, its use in identifying IgAN recurrence appears limited. It is plausible that IgAN recurrence in transplanted kidneys does not result in a significant release of dd‐cfDNA into the bloodstream, compared to what is observed with other pathological processes such as allograft rejection. This could explain the lack of significant differences in %dd‐cfDNA levels between the two transplanted patient groups. The low dd‐cfDNA levels may be attributed to the modest glomerular injury typically seen in recurrent IgAN compared to the more marked histological changes seen in rejection (Akifova et al. 2023).

These observations shed light on the complexities involved in diagnosing and monitoring IgAN recurrence post–kidney transplantation. The recurrence of IgAN in transplanted kidneys presents a challenging scenario, often characterised by insidious disease progression and varying clinical manifestations. Relying solely on %dd‐cfDNA levels may not provide accurate information regarding the presence or absence of IgAN recurrence in kidney transplant recipients.

It is important to note that this study has certain limitations. The relatively small number of participants reduced the statistical power of the analysis, potentially missing subtle differences in %dd‐cfDNA levels between the two patient groups. With a larger cohort, a greater sensitivity in detecting variations in %dd‐cfDNA associated with IgAN recurrence could be achieved.

Another limitation is that this study used a dd‐cfDNA threshold of 0.5% based on previous studies that investigated allograft rejection. However, it is important to note that this threshold is not absolute or definitive. The relationship between allograft injury and dd‐cfDNA levels is likely continuous rather than strictly categorical (Stites et al. 2020).

In this study, recurrent IgAN was defined exclusively based on biopsy‐proven histological confirmation, while the non‐recurrent group did not undergo biopsy due to stable graft function and lack of clinical indication. Although this reflects standard clinical practice, it limits the detection of subclinical recurrence and represents a key study limitation. Future research using protocol (surveillance) biopsies, which are performed at scheduled intervals regardless of clinical status, could address this limitation.

To enhance our understanding and clinical management of IgAN recurrence post‐transplantation, future studies should focus on larger cohorts to validate and refine the use of %dd‐cfDNA as a biomarker. Moreover, conducting longitudinal assessments of %dd‐cfDNA levels over extended periods, starting from pre‐transplantation and continuing post‐transplantation, could provide critical information about disease trajectories and recurrence patterns. Establishing a baseline %dd‐cfDNA level before transplantation would serve as a reference point for monitoring changes and detecting early signs of IgAN recurrence.

## Conflicts of Interest

The authors declare no conflicts of interest.

## Data Availability

The data that support the findings of this study are available from the corresponding author upon reasonable request.
